# Discovering spatiotemporal patterns of COVID-19 pandemic in South Korea

**DOI:** 10.1038/s41598-021-03487-2

**Published:** 2021-12-28

**Authors:** Sungchan Kim, Minseok Kim, Sunmi Lee, Young Ju Lee

**Affiliations:** 1 Department of Applied Mathematics, Kyung Hee University, Yongin, Republic of Korea; 2 Department of Mathematics, Texas State University, San Marcos, TX USA

**Keywords:** Infectious diseases, Applied mathematics

## Abstract

A novel severe acute respiratory syndrome coronavirus 2 emerged in December 2019, and it took only a few months for WHO to declare COVID-19 as a pandemic in March 2020. It is very challenging to discover complex spatial–temporal transmission mechanisms. However, it is crucial to capture essential features of regional-temporal patterns of COVID-19 to implement prompt and effective prevention or mitigation interventions. In this work, we develop a novel framework of compatible window-wise dynamic mode decomposition (CwDMD) for nonlinear infectious disease dynamics. The compatible window is a selected representative subdomain of time series data, in which compatibility between spatial and temporal resolutions is established so that DMD can provide meaningful data analysis. A total of four compatible windows have been selected from COVID-19 time-series data from January 20, 2020, to May 10, 2021, in South Korea. The spatiotemporal patterns of these four windows are then analyzed. Several hot and cold spots were identified, their spatial–temporal relationships, and some hidden regional patterns were discovered. Our analysis reveals that the first wave was contained in the Daegu and Gyeongbuk areas, but it spread rapidly to the whole of South Korea after the second wave. Later on, the spatial distribution is seen to become more homogeneous after the third wave. Our analysis also identifies that some patterns are not related to regional relevance. These findings have then been analyzed and associated with the inter-regional and local characteristics of South Korea. Thus, the present study is expected to provide public health officials helpful insights for future regional-temporal specific mitigation plans.

## Introduction

A novel virus named severe acute respiratory syndrome coronavirus 2 (SARS-CoV-2) was identified as the pathogen for the outbreak of COVID-19 in December 2019^1^. Since then, the COVID-19 pandemic has posed huge challenges to public health officials all around the world. Due to the frequent international flights and human mobility, it took only a few months that COVID-19 spread to more than 200 countries. Currently, many developed countries are in the process of vaccinating their citizens, and some countries hope to soon achieve herd immunity^2^. In fact, the majority of countries with higher proportions of vaccination have shown a significant reduction in the number of COVID-19 cases and deaths from March to June 2021.

Unfortunately, as of July 3, 2021, the confirmed COVID-19 cases have increased worldwide due to the Delta variant^3^. This is one of the new variants of COVID-19 and is a potential threat to the goal of herd immunity. At this point, there are a total of more than 180 million confirmed cases and nearly 4 million deaths in 220 countries^4^. Among others, the US, India, and Brazil are the top three countries of COVID-19 cumulative cases and deaths officially; the US (33,709,176; 605,524), India (30,502,362; 401,050), Brazil (18,687,469; 521,952), respectively. These numbers indicate officially reported cases and may be considerable underestimates due to false negatives^5,6^, lack of tracking systems^7^, and overloading of healthcare facilities^8^. Therefore, it is urgent to understand the spatial–temporal transmission dynamics of COVID-19 to propose effective interventions to mitigate and reduce further morbidity and mortality. Apparently, COVID-19 has disproportionately affected different regional, social, and economic statuses even in developed countries^9–11^. South Korea shows a significant level of variability in the spatiotemporal patterns of COVID-19 as well. As of March 9, 2020, South Korea had a total of 7382 confirmed cases and the largest outbreak of COVID-19 besides China^12^. This was mainly due to few super-spreading events at the Shincheonji Church in Daegu Province and Daenam health care facility in Gyeongsang Province from February 20 to March 20. As of July 3, 2021, the total confirmed cases and deaths of COVID-19 increased to 159,342 and 2025 in South Korea, respectively. The spatial and temporal heterogeneity of COVID-19 has changed over time.

An in-depth understanding of COVID-19 requires the use of mathematical modeling, which has played an essential role to explain complex spatial and temporal transmission dynamics of various infectious diseases. These include recent emerging infectious diseases; novel H1N1 influenza, SARS-CoV-1, Zika, MERS-CoV, and SARS-CoV-2^13^. Recent emerging infectious diseases tend to spread all over the world within a shorter time scale due to dramatic increases in international flights and human mobility^13,14^. There has been much research on spatial–temporal patterns of COVID-19 using various modeling approaches^9,10,15,16^. The spread of COVID-19 during an early stage of the pandemic in South Korea was investigated; 12 significant spatiotemporal clusters were identified and analyzed^17^. They observed that early interventions including 3T (test, trace, treat) were effective so that the cluster size and duration were shortened in time. Castro et al. investigated the spatial and temporal patterns of COVID-19 in Brazil and identified several key factors for failure of region-specific effective interventions^10^. Sartorius et al. employed a Bayesian hierarchical space-time SEIR model to assess the spatiotemporal variability of COVID-19 in England and they examined that mobility and social distancing played a critical role in the spatiotemporal patterns of mobility and mortality^18^. Wang et al. demonstrated the spatiotemporal characteristics and trends of COVID-19 in the United States and the various complex interactions with preventive efforts on COVID-19 were analyzed^19^. Bag et al. explored the spatiotemporal patterns of COVID-19 in India, and further, they examined the interplay between the space-specific patterns and governmental responses^20^.

However, it is very challenging to discover spatial–temporal transmission mechanisms by the standard equation-based framework introduced above. In this work, we propose to discover the high complexity of spatial–temporal dynamics for COVID-19 transmission by employing a data-driven approach based on dynamic mode decomposition. The dynamic mode decomposition method (DMD) originated in the fluid dynamics community as a method to decompose complex flows into spatiotemporal coherent structures. DMD is a matrix-free, data-driven method capable of providing an accurate decomposition of a complex system into spatial–temporal coherent structures that may even be able to predict the short-time future state. Since Schmid and Sesterhenn^21^ first introduced the DMD algorithm and demonstrated its ability, there have been tremendous works in DMD, and DMD became even more popular and is still in development today. This includes a sparsity-promoting DMD^22^, a randomized DMD^23^, which scales with the intrinsic rank of the dynamics, a consistent DMD, a new method for computing DMD operator based on a variational framework^24^. DMD has been successfully used for computational epidemiology^25^. Bistrian et al.^26^ proposed a framework for reduced-order modeling and forecasting of non-intrusive data with application to epidemiology, using a technique based on randomized DMD combined with ARIMA (AutoRegressive Integrated Moving Average)^27^ and this has been used also for modeling of SARS-CoV-2 dynamics obtained from the raw data reported by World Health Organization^28^. Proctor et al.^29^ have demonstrated how DMD can aid in the analysis of spatial–temporal disease data. It is shown that DMD is an effective and efficient computational analysis tool for the study of infectious disease taking into account several tests’ data such as Google Flu Trends data, pre-vaccination measles in the UK, and paralytic poliomyelitis wild type-1 cases in Nigeria. We note though that in particular, Google Flu Trends data is shown to be overall more influenced by the media clamor than by true epidemiological burden as studied in^30,31^.

In this paper, we propose a compatible window-wise dynamic mode decomposition (CwDMD). The notable difference of our work from other available works is that we tackle COVID-19 time series data in a way that the data sets are made to be consistent in the sense of Tu et al.^32^. Basically, the compatible window is a selected the data set that can be modeled by a linear operator, thereby making DMD analysis meaningful. Further, we show that the consistency is equivalent to the linearity and demonstrate that DMD produces misleading data interpretation for inconsistent or nonlinear data in general. This indicates that the direct and reliable DMD analysis of large time-series data such as COVID-19 data is not feasible. We develop a strategy to choose an adequate set of representative subdomains called windows in which an appropriate balance or compatibility between spatial and temporal resolutions is built. The total size-times duration of all the windows serving a given system depends only on local situations that can arise in the full time-series data. We then apply DMD to each window that results in robust and reliable data analysis. It is easy to see that if the data is linear, DMD analysis will be adequate while it is not for nonlinear data. Oftentimes such an inadequacy has been justified through the Koopman mode analysis in the framework of Hankel DMD. However, it is well-known that Hankel DMD is proven to work only for ergodic data^33,34^. These frameworks, therefore, can not be applied in general, for highly nonlinear data. Such data includes internal solitary wave as discussed in^35^ as well as COVID-19 data analyzed in the present paper, which are not necessarily ergodic. It is notable that a recent work by Zhang et al.^35^ is closely relevant to our method. However, their work is not based on compatible windows, i.e., the choice of windows is constructed without respecting the consistency. Phase studies are not investigated either unlike the proposed study in this paper. Furthermore, we make significant and novel progress from the consistency assumption that the data fitting for any given window can be achieved accurately only by finding the coordinate of any single data within the window in terms of DMD modes. This allows us to achieve a significant computational reduction. The identified coordinate is then used as a certain scale for the selection of important DMD modes.

Our new method is used to investigate the spatiotemporal patterns of COVID-19 in South Korea from January 20, 2020 to May 10, 2021. A total of four compatible windows have been selected from the given COVID-19 time series data. The spatiotemporal patterns of these four windows are then analyzed by a few important DMD modes selected based on our new criterion. Several hot and cold spots were identified, their spatial–temporal relationships, and some hidden regional patterns were discovered. Our analysis reveals that the first wave was contained in the Daegu and Gyeongbuk area, but it spread rapidly to the whole of South Korea after the second wave. Later on, the spatial distribution is seen to become more homogeneous after the third wave. These findings have then been associated with the inter-regional and local characteristics of South Korea. We expect that the present study can provide public health officials helpful insights for future regional-temporal specific mitigation plans.

## Results

### Spatial–temporal characteristics of COVID-19 in South Korea

**Figure 1:**
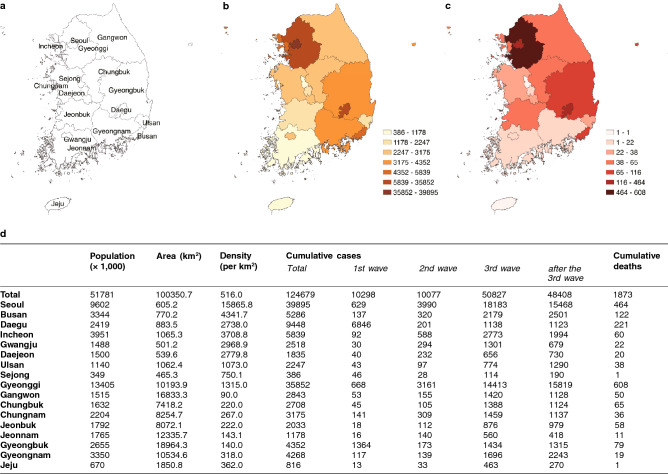
Spatial distribution of the cumulative confirmed and deaths of COVID-19 as of May 10, 2021. (**a**) A map of South Korea. South Korea is divided into 17 first-tier administrative divisions: 7 metropolitan cities (Seoul, Busan, Daegu, Incheon, Gwangju, Daejeon, and Ulsan), 1 special self-governing city (Sejong), and 9 provinces. The metropolitan area refers to Seoul, Incheon, and Gyeonggi. (**b**) Cumulative confirmed cases. (**c**) Cumulative deaths. Geographical descriptions such as population, area, and population density of each region; and COVID-19 profiles are in (**d**). Population density between metropolitan cities and non-metropolitan areas is extremely polarized, except Gyeonggi. The total population of three metropolitan areas is about 26 million as of May 2021, which is more than 50% of the South Korean population.

**Figure 2:**
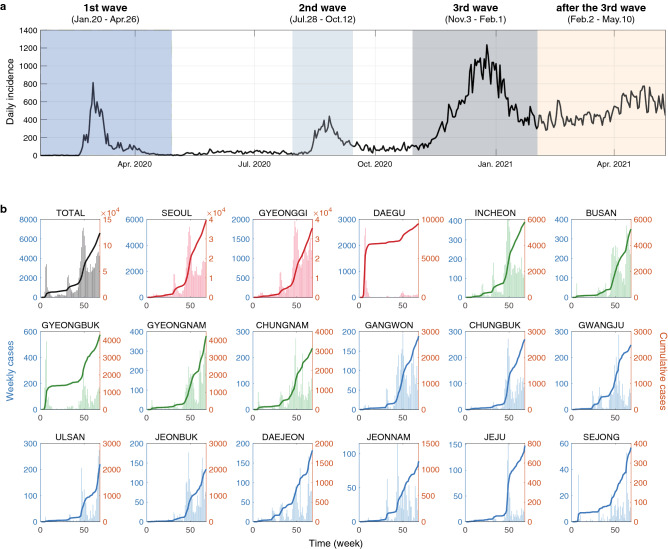
Time series of COVID-19 outbreak in South Korea. (**a**) Daily incidence of COVID-19 in South Korea. South Korea went through three big waves, after the third wave, the incidence has been maintained with no significant increase or decrease. The four windows of main interest were colored and given as; (1) the first wave (January 20, 2020–April 26, 2020); (2) the second wave (July 28, 2020–October 12, 2020); (3) the third wave (November 3, 2020–February 1, 2021); and (4) after the third wave (February 2, 2021–May 10, 2021). (**b**) Weekly incidence and cumulative cases in 17 regions, plotted as the bars and as a curve, respectively. The three highest cumulative cases, the next five highest cases, and the rest cases are marked with red, green, and blue, respectively.

In this section, we present an overview of COVID-19 data collected in South Korea (see Fig. 1 for more description). Daily confirmed cases and deaths of COVID-19 from January 20, 2020 to May 10, 2021, were obtained from the Korea Centers for Disease Control and Prevention (KCDC) and each provincial website^12^. As of May 10, 2021, there were a total of 127,772 COVID-19 confirmed cases and 1875 deaths in South Korea. To analyze the spatiotemporal patterns of COVID-19, the spatial distribution of COVID-19 confirmed cases is refined in 17 first-tier administrative divisions of South Korea. Figure 1 shows a South Korea map (a) with spatial distributions of the cumulative number of COVID-19 confirmed cases (b) and the cumulative number of COVID-19 deaths (c). As displayed in b, c, d of Fig. 1, South Korea shows a high level of spatial and temporal heterogeneity in 17 regions. We can observe that the main characteristics of the temporal patterns of South Korea can be placed into the particular four stages, i.e., three big waves and the last stage. More precisely, the first window is from January 20, 2020 to April 26, 2020, the second window is from July 28, 2020 to October 12, 2020, the third window is from November 3, 2020 to February 1, 2021, and the period after the third wave is February 2, 2021, to May 10, 2021. These are chosen as four windows and represented by different colors in Fig. 2a.

The first case of COVID-19 in South Korea was a 35-year-old Chinese woman who traveled from Wuhan, China, and was confirmed on January 20, 2020. She entered the Incheon international airport and she was isolated at a hospital upon entry. After the index case, only 30 confirmed cases have occurred until February 17, 2020. However, there was an explosive outbreak in Daegu due to the superspreading events from the Shincheonji Church-related clusters from February 18 to March 23, 2020^12^. As a result, the first wave (January 20, 2020–April 26, 2020, see Fig. 2a) was focused on the Daegu and Gyeongbuk area with almost 80% of a total of 10,298 cases (Daegu 6846 and Gyeongbuk 1364). Since March 2020, the epicenter of COVID-19 has begun to move from the Daegu and Gyeongbuk area to Seoul and Gyeonggi regions. A few sporadic clusters of COVID-19 continued in Seoul including the Guro call center and the Itaewon club cluster in May 2020. From July 28 to October 12, 2020, the second wave started in Seoul and Gyeonggi Province (see Fig. 1d). The main cause of the second wave was the rally held at Gwanghwamun Square in Seoul. Seoul city has the highest in the confirmed cases and Gyeonggi Province has the second-highest in the confirmed cases and the highest in deaths. The largest wave was the third wave from November 3, 2020 to February 1, 2021. This was partly due to the winter seasons, which results in a favorable condition for close contact between people staying indoors. After the third wave, the constant level of COVID-19 cases has been maintained nationwide from February 2, 2021, to May 10, 2021.

### Analysis of spatial–temporal COVID-19 in South Korea

In this section, we shall present the analysis of spatial–temporal COVID-19 in South Korea. Figure 2 displays confirmed cases of COVID-19 in South Korea from January 20, 2020, to May 10, 2021. Panel a of Fig. 2 shows the daily confirmed cases while the panel b of Fig. 2 illustrates region-specific COVID-19 weekly confirmed cases (bars on the left) and cumulative cases (solid curves on the right) for 17 first-tier administrative divisions of South Korea. The three highest cumulative cases, which include Seoul, Gyeonggi, and Daegu are marked in red, the next five highest cases are marked in green, and the rest of the cases are marked in blue.

First of all, Supplementary Fig. S1 displays the evolution of spatial distributions in 17 regions; the top panels show the cumulative number of COVID-19 cases per 100,000 on the last day of each period. The bottom panels show the cumulative number of COVID-19 cases per 100,000 during each period. The bottom panels indicate that the hot spots were moving from Daegu to Seoul and Gyeonggi while Jeonnam remained the cold spot in the first, third, and last periods. Interestingly, Daegu and Gyeongbuk were the cold spots during the second wave after the severe first outbreak.

The chosen four windows are then used to apply CwDMD, which results in the discrete DMD modes and eigenvalues in each window. Supplementary Figs. S2–S5 compare the results of the DMD data fitting with the region-specific COVID-19 data for each window. There is a perfect agreement between the COVID-19 data (red dot) and the DMD output (black solid) in all 17 regions.

CwDMD has been used to investigate the spatiotemporal pattern of COVID-19 in 17 regions, whose discussions are presented in the following four subsections. Note that a few important DMD modes selected in each window are categorized into three regimes, oscillatory, growing, and decaying. These are then used for the phase and magnitude analysis of each window.

#### The first wave

The first wave is chosen as the total of 14 weeks and so, the spatial vs temporal resolution is 17 to 14. This is compatible as discussed in the section for “Methods”.

**Figure 3:**
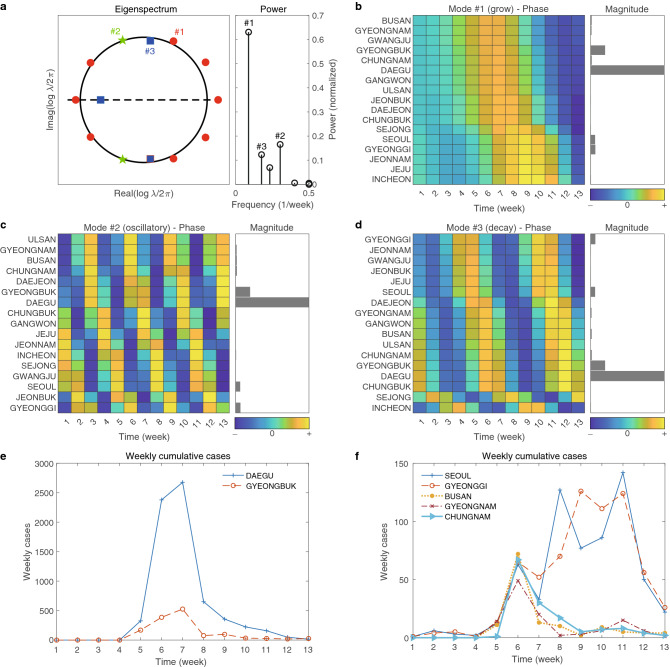
The first wave: DMD eigenvalues and modes. (**a**) shows eigenspectrum $\{\lambda _j\}_{j=1,\cdots }$ in the left and powers, defined as $\{|\lambda _j^p| \Vert \alpha _j \phi _j \Vert _F\}_{j=1,\cdots }$, in the right. The first three DMD modes that represent growing, oscillatory and decaying modes are enumerated as $\#1$, $\#2$, and $\#3$. (**b**–**d**) Show the phase and magnitude of the selected DMD modes, $\#1$, $\#2$, and $\#3$, respectively. (**e**) and (**f**) show time series of weekly cumulative cases for some selected regions. (**e**) is for high transmission areas, Daegu and Gyeongbuk, while (**f**) is for other relatively low transmission areas.

In Fig. 3, we show the power of DMD modes in a, i.e., the measure of the scaled size of the DMD modes (see the section of “Methods” in details). The power is used for the selection of dominant DMD mode and the selected DMD mode is then utilized for both magnitude and phase analysis.

We note that the first three DMD modes of the highest power were chosen and they are denoted by #1, #2, and #3 as shown in Fig. 3a. In fact, the selected three DMD modes correspond to the growing, the oscillatory, and the decaying modes, respectively in the discrete dynamical system for the first window. The magnitude analysis has been performed using these dominant DMD modes. We observe that all three DMD modes show that Daegu and Gyeongbuk have the largest magnitude and Seoul and Gyeonggi are next. These are indicated by gray bars in b, c, d, respectively, and consistent with the cumulative confirmed cases of the first wave given in e and f of Fig. 3.

Next, we explored the phase analysis from the three selected DMD modes. We note that phase or phase difference can be interpreted as the time (in week) between peak to peak of the region-specific COVID-19 outbreak. Namely, the smaller the phase difference of two different regions is, the closer the peaks of these regions will be. We find that in all three DMD modes, the phases of Busan, Gyeongnam, and Chungnam are similar. Note that these three regions are close to the epicenter. Consequently, we find a strong correlation between the phase of the southern part of South Korea and the distances from the epicenter, i.e., Daegu and Gyeongbuk. This is consistent with the data presented in f of Fig. 3.

On the other hand, the phase of DMD mode #1, shows that there is a time lag of 2–3 weeks between the peaks of Seoul and Gyeonggi and those from Busan, Gyeongnam, Gyeongbuk, and Daegu. In particular, from the fact that the DMD mode #1 is a growing mode, the above conclusion indicates that there was definitely a different cause for the COVID-19 outbreak of Daegu and Gyeongbuk from that of Seoul and Gyeonggi. Note that it can be clearly identified in the graph of e and f in Fig. 3. More precisely, the weekly confirmed cases of Seoul and Gyeonggi are similar to those of other regions from weeks 5 to 7. However, the confirmed cases began to increase from week 8 to 13, while those of other regions decreased. We later found that this peculiar behavior could be associated with a few large workplace-related clusters such as the Guro-Gu call center in Seoul and Gyeonggi from March 2020 to April 2020^36,37^.

#### The second wave

The second wave is chosen as the total of 11 weeks and so, the spatial vs temporal resolution is 17 to 11. This is compatible as discussed in the section for “Methods”.

**Figure 4:**
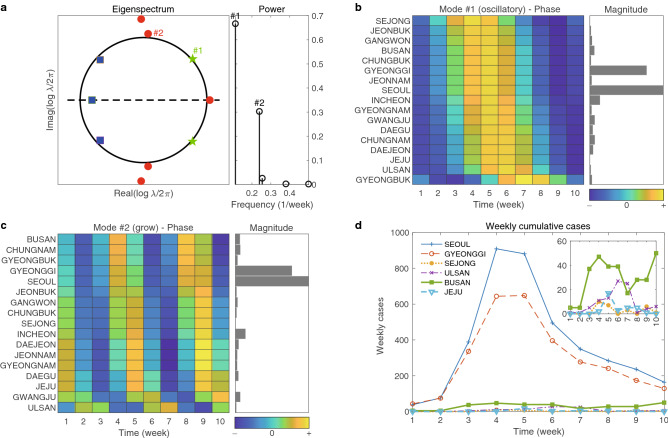
The second wave: DMD eigenvalues and modes. (**a**) shows eigenspectrum $\{\lambda _j\}_{j=1,\cdots }$ in the left and powers, defined as $\{|\lambda _j^p| \Vert \alpha _j \phi _j \Vert _F\}_{j=1,\cdots }$, in the right. The first two DMD modes of highest powers are enumerated as $\#1$ and $\#2$. (**b**) and (**c**) show phase and magnitude of the $\#1$ and $\# 2$ DMD modes, respectively. In the phase diagram, regions, whose phases are similar are gathered. (**d**) is the time series for weekly cumulative cases for some selected regions.

In Fig. 4, we show the power of DMD modes in a. The power is used for the selection of dominant DMD mode and the selected DMD mode is then utilized for both magnitude and phase analysis. We note that the first two DMD modes of the highest power were chosen and they are denoted by #1 and #2 as shown in Fig. 4a. The selected two DMD modes correspond to the oscillatory and the growing modes, respectively. The magnitude analysis has been performed using these dominant DMD modes. The weekly confirmed cases of the total of six selected regions are then shown in d of Fig. 4.

The magnitude analysis using both of these selected DMD modes shows that Seoul and Gyeonggi have a significantly large magnitude of confirmed cases. This is in fact, consistent with the data shown as Fig. 4d. The main drive behind this large magnitude can be correlated with the outbreak from the rally held at Gwanghwamun Square in Seoul on August 15, 2020. Note that this rally was organized by SarangGeil Church in Seoul and people from all regions of South Korea participated. We observe that, unlike the first wave, the magnitude of Daegu and Gyeongbuk are relatively small. This can be attributed to the continued strict mitigation interventions in the Daegu and Gyeongbuk area since the first wave. See also the COVID-19 cases shown in Fig. 1 as well as in Supplementary Fig. S1, which are consistent with our magnitude analysis for Daegu and Gyeongbuk.

We now explore the phase analysis from the two selected DMD modes. First, we begin with the following facts; (1) the maximum phase difference in the DMD mode #1 is between Sejong and Ulsan and its value is 1.54 weeks; (2) the maximum phase difference is 1.04 weeks and it is between Busan and Jeju in the DMD mode #2. The relatively short phase difference indicates that the second wave can be characterized as an almost simultaneous nationwide spread. This can be attributed to the fact that all participants from all regions who attended the rally in Seoul returned to their home region within a few days, i.e., less than a week^38^.

#### The third wave

The third wave is chosen as the total of 13 weeks and so, the spatial vs temporal resolution is 17 to 13. This is compatible as discussed in the section for “Methods”.

**Figure 5:**
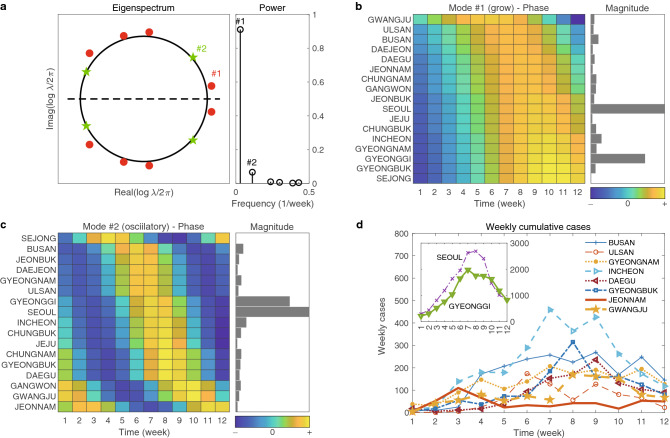
The third wave: DMD eigenvalues and modes. (**a**) shows eigenspectrum $\{\lambda _j\}_{j=1,\cdots }$ in the left and powers, defined as $\{|\lambda _j^p| \Vert \alpha _j \phi _j \Vert _F\}_{j=1,\cdots }$, in the right. The first two DMD modes of highest powers are enumerated as $\#1$ and $\#2$. (**b**) and (**c**) show the phase and magnitude of the $\#1$ and $\# 2$ DMD modes, respectively. In the phase diagram, regions, whose phases are similar are gathered. (**d**) is the time series for weekly cumulative cases for some selected regions.

In Fig. 5, we show the power of DMD modes in a. The power is used for the selection of dominant DMD mode and the selected DMD mode is then utilized for both magnitude and phase analysis. We note that the first two DMD modes of the highest power were chosen and they are denoted by #1 and #2 as shown in Fig. 5a. The selected two DMD modes correspond to the growing and the oscillatory modes, respectively. The magnitude analysis has been performed using these dominant DMD modes. The weekly confirmed cases of the total of eight selected regions are then shown in d of Fig. 5.

The magnitude analysis using both of these selected DMD modes shows that Seoul and Gyeonggi have a significantly large magnitude of confirmed cases, similar to the second wave. This is in fact, consistent with the data shown as Fig. 5d. This is due to the cold winter seasons, as people favorably stayed indoors for close contacts, which is enhanced by the higher population density in Seoul and Gyeonggi; the South Korean population is highly disproportionate and the metropolitan area has more than 50% of the total South Korean population.

The phase analysis in this wave shows that the maximum phase difference is larger than that of the second wave for both modes. Namely, the maximum phase difference in the DMD mode #1 is 4.02 weeks, which is between Busan and Jeonnam, while the maximum phase difference in the DMD mode # 2 is 4.13 weeks, which is between Gyeonggi and Jeonnam. In particular, regions grouped according to the higher phase similarity are (1) Busan, Gyeongnam, and Ulsan, which are all located in the southeast area, (2) Seoul, Gyeonggi, and Incheon, which are all located in the northwest area, and (3) Daegu and Gyeongbuk, which are at the central area. This analysis identifies that there are strong spatial correlations in the third wave. This seems to be natural. But, to our surprise, we observe that there is more or less independent phase behavior between Gwangju and Jeonnam in DMD mode # 2. This means that Jeonnam is not much affected by the outbreak of COVID-19 in Gwangju, even if Jeonnam surrounds Gwangju. In fact, it is in this way throughout the whole time when COVID-19 data is collected. This indicates that the expected spatial correlation is sometimes misleading. Additionally, the similar phenomenon is also observed between in Daejeon and Chungnam.

#### The period after the third wave

The period after the third wave is chosen as the total of 13 weeks and so, the spatial vs temporal resolution is 17 to 13 again like the third wave. The main feature of this period is that the weekly incidence is relatively large all over South Korea.

**Figure 6:**
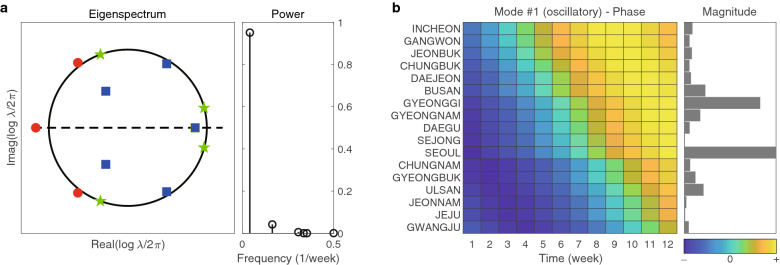
After the third wave: DMD eigenvalues and modes. (**a**) shows eigenspectrum $\{\lambda _j\}_{j=1,\cdots }$ in the left and powers, defined as $\{|\lambda _j^p| \Vert \alpha _j \phi _j \Vert _F\}_{j=1,\cdots }$, in the right. The first two DMD modes of highest powers are enumerated as $\#1$ and $\#2$. In this wave, the only one DMD mode of the dominant power is selected. (**b**) shows the phase and magnitude of the selected DMD mode. In the phase diagram, regions, whose phases are similar are gathered.

In Fig. 6, we show the power of DMD modes in A. The power is used for the selection of dominant DMD mode and the selected DMD mode is then utilized for both magnitude and phase analysis. We note that a single DMD mode shows the dominant power and so, only this DMD mode is chosen and denoted by #1 as shown in Fig. 6a. The selected DMD mode corresponds to the oscillatory mode.

The magnitude analysis using this selected DMD mode shows that Seoul and Gyeonggi have the largest magnitude, which is consistent with the highest cumulative COVID-19 cases during the period after the third wave in these regions as shown in Fig. 2b. This consistency also holds for the next largest magnitudes or cumulative cases occurring in the southeast areas, which include Busan, Ulsan, and Gyeongnam.

The phase analysis in this period shows that the maximum phase difference is 11.8 weeks, which is from Incheon and Gwangju. Furthermore, the phase difference between neighboring regions such as Seoul and Gyeonggi, Daejeon and Chungnam, and Daegu and Gyeongbuk is also more than three weeks, which is relatively large. This indicates that overall large weakly incidence in each region is local in nature. Namely, the outbreaks in each region are mainly due to local outbreaks within the region and the inter-regional correlation of outbreaks seems to be irrelevant in this period. This has been further justified by investigating the spatial variations using the estimation of so-called the coefficient of variation below.

### Time dependent spatial variation of COVID-19 in South Korea

In this section, we further investigate the data to quantify the time-dependent spatial variation of COVID-19 in South Korea over the period of interest.

**Figure 7:**
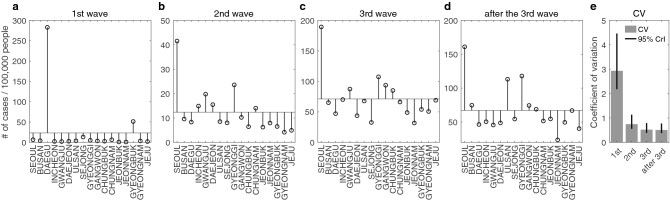
Regional variation in infection rate relative to the average rate. (**a**–**d**) show the rate of incidence per 100,000 people relative to the average rate for the first wave, second wave, third wave and after the third wave, respectively. The only incidence rates of Daegu and Gyeongbuk are shown to be higher than average. After the first wave, the incidence rates of Daegu and Gyeongbuk decreased to be under the average. The coefficient of variation (CV), defined as the ratio of the standard deviation to the mean is plotted in (**e**). Grey bar and black vertical line represent the CV of each period and its 95% credible interval (CrI), respectively. The CV estimated for each period is found to be 2.93 (95% CrI: 2.19–4.47), 0.75 (0.56–1.14), 0.52 (0.39–0.79), and 0.51 (0.38–0.77), respectively. This result shows that regional variation in the rate of incidence per 100,000 population becomes gradually uniform over time.

We investigate the rate of incidence per 100,000 people in each region for the first wave, the second wave, the third wave, and the period after the third wave and plot this in Fig. 7a–d, respectively. This shows that the regional variation in weekly incidence is gradually decreasing over time. We observe that in the first wave (see Fig. 7a), only the rate of incidence for Daegu and Gyeongbuk is shown to be higher than average. After the first wave, the rate of incidence for Daegu and Gyeongbuk becomes below the average, whereas that of Seoul and Gyeonggi stays higher than the average. Even if it is not definitely clear, as time proceeds, the regional differences seem to get smaller. To quantify this observation on the time-dependent regional difference in the incidence rate, we compute so-called the coefficient of variation (CV) for the rate of incidence per 100,000 people. The CV is defined by the ratio of the standard deviation to the mean^39^. This is a dimensionless number that can be used to compare the dispersion of groups with different means or different units. Similar to the standard deviation, the larger the CV is, the more over-dispersed the data will be. The computed CV is presented in Fig. 7e, in which we find that the CV decreases in time. More precisely, we have 2.93 CV (95% credible interval (CrI): 2.19–4.47 CV) for the first wave, 0.75 CV (95% CrI: 0.56–1.14 CV) for the second wave, 0.52 CV (95% CrI: 0.39–0.79 CV) for the third wave, and 0.51 CV (95% CrI: 0.38–0.77 CV) for the period after the third wave. This result clearly demonstrates that the first drastic reduction in CV occurred during the second wave, and the regional variation of weekly incidence tends to decrease over time. Namely, the spatiotemporal incidence pattern tends to be homogeneous, thereby indicating that the local outbreaks are dominant in most of the regions for the period after the third wave.

### Novel compatible window-wise dynamic mode decomposition

**Figure 8:**
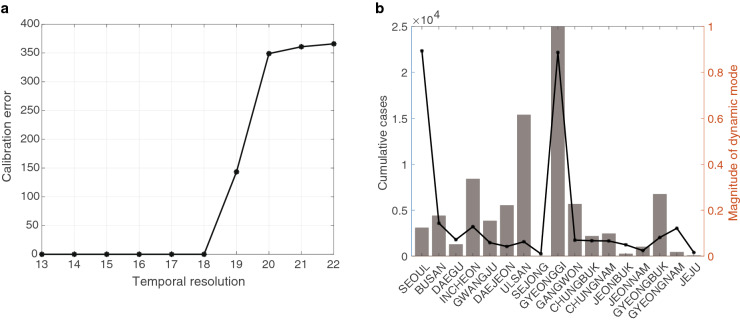
Results showing the inadequacy of the standard DMD applied to incompatible data. (**a**) shows the calibration error as a function of temporal resolutions. The spatial resolution is fixed as 17 and we see that as the temporal resolution becomes larger than 18, the calibration errors start to increase. In (**b**), we consider the total of 19 weeks’ time series data from Dec. 27, 2020–May 8, 2021, which is incompatible with 17 spatial resolution. We selected DMD mode consistently and analyzed its magnitude. Clearly, (**b**) shows that the magnitude does not adequately represent that of data. Recall Figs. 5 and 6 are for the compatible windows, in which such erroneous result do not occur.

Our data analysis using CwDMD has clearly shown the usefulness of the method to identify patterns of the spatially and temporally correlated nonlinear data. It is shown as well that some hidden patterns could be identified. The standard DMD, however, has a limitation in that it may provide misleading analysis generally for the inconsistent data^32^. The inconsistent data, equivalent to the nonlinear data can be interpreted as the data in which spatial resolution, the amount of spatial detail is given incompatible with the temporal resolution, the amount of temporal detail. Precise condition for the compatibility is obtained in section for “Methods”. In Fig. 8 we have considered the COVID-19 time series data collected in a total of 17 regions. The standard DMD operator is shown to be able to fit the data perfectly in case a total of 18 or smaller temporal data is selected. The number 18 is the maximal time resolution for which the compatibility between spatial and temporal resolutions is valid. As the temporal resolution increases, the data fitting quality by DMD deteriorates significantly. This is unequivocally interpreted that DMD is inadequate to provide meaningful data analysis for these cases. To quantify the inadequacy, we investigate the phase and magnitude analysis from the selected DMD mode. For 19-week data from December 27, 2020–May 8, 2021, there is an evident disagreement between the COVID-19 data (black solid) and the DMD output (orange bar). The actual data indicates that the number of confirmed cases is higher in Gyeonggi and Seoul and it is relatively lower in Ulsan. However, DMD data analysis indicates otherwise that the number of confirmed cases in Ulsan is higher than in Seoul. This implies that the selected DMD mode does not represent the data pattern adequately. Thus, the direct and reliable DMD analysis of large time-series data is concluded not to be feasible unless it is linear.

We, therefore, arrive at the need of introducing a novel compatible window-wise dynamic mode decomposition. The main issue in DMD for large time-series lies in the nonlinearity of the data. The point of CwDMD is that for any given nonlinear data, it is proven to be possible to select an adequate set of representative subdomains called windows, each containing moderate-sized linear data. For example, Fig. 2a, shows specially chosen windows for COVID-19 data in South Korea we analyze. The total size-times duration of all the windows serving a given system depends only on local situations that can arise in the full-time series data. We then apply the standard DMD for each window. This strategy is called the compatible window-wise dynamic mode decomposition (CwDMD). Basically, CwDMD is a collection of DMD for a specially selected set of consistent windows. In each window, we choose the most significant DMD modes, and the reconstructed data in its dimension, from the selected DMD modes are constructed and investigated to understand the actual data.

## Discussion

In this study, we have developed a novel data-driven framework: *compatible window-wise dynamic mode decomposition* (CwDMD). Using the CwDMD, we have identified the spatiotemporal transmission patterns of COVID-19 in South Korea from January 20, 2020 to May 10, 2021. It is generally very challenging to uncover COVID-19 transmission dynamics since there exists a complex interplay among various time-varying factors such as virus, human, mobility, socio-economic infrastructures, and public health policies. However, our CwDMD analysis successfully elucidates how spatial correlations among 17 regions evolve in the presence of such complex features.

The first wave was focused on the Daegu and Gyeongbuk area, which was mainly caused by the superspreading events from the Shincheonji Church-related clusters^12,17^. It spread to several regions nearby, but this was quickly contained. This was due to aggressive interventions such as drive-through or walk-through rapid PCR testing, contact tracing, isolation, mask distribution, and social distancing^40^. Most of all, the behavior and awareness of South Koreans were the most crucial reasons for great success. As a result, the substantially largest outbreaks from the Shincheonji Church-related clusters did not last for more than a month. Our analysis also confirmed that the local outbreaks were kept in the Daegu and Gyeongbuk areas. Towards the end and after the first wave, a few major large-scale outbreaks occurred in the metropolitan regions including Seoul, Gyeonggi, and Incheon. For example, in Seoul, there were a few sporadic large outbreaks, which include the Guro-call center, Itaewon Club, and Richway (Seoul-based health product retailer) between March 2020 and June 2020.

Execution of online school, which was initiated in the middle of the first wave, and ongoing intensive interventions contributed to maintaining a low level of COVID-19 outbreaks nationwide until the rally held in Seoul. On August 15, 2020, the rally led by SarangGeil Church caused 641 cases in Seoul and thus initiated the second wave. Our phase analysis for the second wave captured that COVID-19 spread rapidly throughout the nation. This is linked to the fact that people participated in the rally and returned to their home regions in a few days^38^, which arose local outbreaks in every region as well.

The local outbreaks became dominant compared to inter-regional outbreaks during the winter season from November 2020 to February 2021. The large-scale local spread of COVID-19 led to the third wave with the largest cumulative cases nationwide. Since the outbreaks were significantly severe in the third wave and the majority of the cases were focused on the metropolitan area, region-specific public health policies were first implemented and risk assessment level for social distancing interventions was refined from Level 3 to Level 5, as of November 7, 2020^41^. Moreover, region-specific restrictions of large gatherings, such as prohibiting gatherings of more than four people and closing shops after 10 pm, have been imposed during the third wave^42^ as well. Additionally, a special quarantine period was imposed on Thanksgiving and the New Year’s holidays nationwide. These strict interventions combined with vaccination have slowed down the third wave from February 2021. Vaccination started from February 26, 2021, with a slow rate at the early stage; 7.1% of the primary dose; 1.1% of the second dose, as of May 10, 2021^2^. COVID-19 has then been maintained without major outbreaks for more than four months after the third wave, between February 2, 2021 to May 10, 2021.

Overall, cumulative cases and deaths of COVID-19 in South Korea seem not that large compared to those of other countries with similar population densities, and the duration of each wave seems not too long either. For example, as of July 9, 2021, a total of 814,533 cumulative cases and 14,933 deaths of COVID-19 in Japan were reported while a total of 165,344 cumulative cases and 2036 deaths in South Korea^4^ were reported. Japan’s vaccination rate (2.1 % of the primary dose and 1.0% of the second dose as of May 10, 2021) and population density (337/${\mathrm{km}}^2$) are similar to those of South Korea. However, the fourth big wave occurred in Japan, from March 2021 to May 2021 with a maximum daily number of confirmed cases of more than 6000. This can be associated with the fact that Japan imposes voluntary social distancing policy, while South Korea continues to enforce compulsory social distancing policies even after the third wave. Japan has invoked a number of COVID-19 State of Emergencies, but compulsory policies such as forced suspension or lockdown was not imposed^43^. On the other hand, policies in South Korea such as prohibiting gatherings of more than four people and closing shops after 10 p.m. forcibly prevent further infections from occurring. It is worth mentioning that there are data-related issues in this study. First, the official (reported) data could be different from the real ones due to the selective biases of various kinds^5,6^. Next, other factors such as temperatures, seasonality, UV radiation, pollution, etc.^44–46^ are not included in the analysis.

South Korea is one of the most successful countries for mitigating and preventing the COVID-19 pandemic. Since South Korea has learned a valuable lesson from the MERS-CoV outbreak, which was the largest outbreak originated from the Middle Eastern countries in 2015, various preparedness plans have been initiated for emerging infectious diseases including medical infrastructure and transparent data disclosure through daily briefings^47^. Real-time infection transmission notification through mobile phone applications or websites, and a real-time alarm system through mobile phone (including location-specific risk notification through GPS) have been newly developed during the COVID-19 pandemic. In addition, South Koreans were quickly alert and carried out voluntary preventing activities such as wearing a mask and prohibiting gatherings. With such an ensemble of national infrastructure and citizens’ voluntary participation in quarantine, South Korea demonstrates its superiority in handling COVID-19 outbreaks through successful mitigation strategies.

DMD has been successful to extract spatial–temporal coherent patterns in a specific form of periodic, growing, and decaying dynamical spectrum decomposition^34^. On the other hand, it is shown that balance between spatial and temporal resolutions has to be taken into account since otherwise, DMD mode analysis can result in erroneous data interpretation for highly nonlinear time series data. This balance is mathematically identified as the linearity of data in this paper, which means that DMD can in general make sense only for the appropriate selection of windows from the full temporal data sets so that spatial resolution is larger than the temporal resolution. This clearly generates the limitation of the use of classical DMD and/or its variants^22,25^ since oftentimes it is useful to extract spatiotemporal patterns for rather long data sets. To overcome this issue, one can select a special set of the time series data with certain labels as discussed in^48^ or more generally, one can use a certain multiscale temporal representation of the data. Namely, one can decompose the temporal steps, from fine to coarse so that in coarse level, the global data makes the linearity, while the fine-scale is handled only in several local windows. Somewhat similar but different idea, named as multiresolution DMD can be found at^49^. Overall, a systematic method or mathematical modeling for forecasting COVID-19 data is an open and challenging issue. The multiscale approach briefly described above is potentially useful to generate the prediction operator. Lastly, if we can identify the data related to external controls and interventions to stop spreading COVID-19, then we may be able to apply DMD with control, presented in^50^ for analysis, which is yet to be investigated.

## Methods

### Compatible window-wise dynamic mode decomposition (CwDMD)

In this section, we shall describe the compatible window-wise Dynamic Mode Decomposition (CwDMD), a novel dynamic mode decomposition method that respects the compatibility of the data set. A detailed statement of compatibility will be presented as well. Basically, we present a new observation that the consistent data is a linear data and suggest that DMD has to be applied for the consistent or linear data. A compatibility condition is a way of achieving this consistency or linearity of the data set. We shall show that certain windows of the given time series data has to be selected so that a balance between the spatial and temporal resolution of the data set is made. This balance will then lead to the linearity of the selected windows. The application of DMD for each window is shown to result in accurate data analysis.

Throughout this section, for the sake of convenience, we denote ${\mathbb {C}}^{n\times \ell }$ by the space of complex matrices of size $n\times \ell$. For $n = 1$ or $\ell = 1$, we shall omit writing it. Namely, for $\ell = 1$, we set ${\mathbb {C}}^n := {\mathbb {C}}^{n \times 1}$, that of which is sets of complex vectors of size *n*. For any element $c \in {\mathbb {C}}$, we shall denote ${\overline{c}}$ by its complex conjugate. We shall denote $\mathop {\cdot }\limits _{\sim }$ by the vector and $$\mathop {\cdot }\limits _{\begin{array}{c} \approx \end{array}}$$ by the tensor. For $$\mathop {M}\limits _{\begin{array}{c} \approx \end{array}} \in {\mathbb {C}}^{n\times \ell }$$, its null and range will be denoted by $${\mathcal {N}}(\mathop {M}\limits _{\begin{array}{c} \approx \end{array}})$$ and $${\mathcal {R}}(\mathop {M}\limits _{\begin{array}{c} \approx \end{array}})$$, respectively. We denote $$\mathop {\mathop {M}\limits _{\begin{array}{c} \approx \end{array}}}\nolimits ^{*}$$ by its complex adjoint matrix, and also denote $$\mathop {\mathop {M}\limits _{\begin{array}{c} \approx \end{array}}}\nolimits ^\dag $$ by the pseudoinverse of $$\mathop {M}\limits _{\begin{array}{c} \approx \end{array}}$$. The symbol $$\mathop {\delta }\limits _{\begin{array}{c} \approx \end{array}}$$ denotes the identity matrix. Note that $$\mathop {\mathop {M}\limits _{\begin{array}{c} \approx \end{array}}}\nolimits ^\dag $$ satisfies the following conditions:

$$\begin{aligned} \mathop {M}\limits _{\begin{array}{c} \approx \end{array}} \mathop {\mathop {M}\limits _{\begin{array}{c} \approx \end{array}}}\nolimits ^\dag \mathop {M}\limits _{\begin{array}{c} \approx \end{array}} = \mathop {M}\limits _{\begin{array}{c} \approx \end{array}}, \,\, \mathop {\mathop {M}\limits _{\begin{array}{c} \approx \end{array}}}\nolimits ^\dag \mathop {M}\limits _{\begin{array}{c} \approx \end{array}} \mathop {\mathop {M}\limits _{\begin{array}{c} \approx \end{array}}}\nolimits ^\dag = \mathop {\mathop {M}\limits _{\begin{array}{c} \approx \end{array}}}\nolimits ^\dag , \,\, (\mathop {M}\limits _{\begin{array}{c} \approx \end{array}} \mathop {\mathop {M}\limits _{\begin{array}{c} \approx \end{array}}}\nolimits ^\dag )^* = \mathop {M}\limits _{\begin{array}{c} \approx \end{array}} \mathop {\mathop {M}\limits _{\begin{array}{c} \approx \end{array}}}\nolimits ^\dag , \,\, \text{ and } \,\,(\mathop {\mathop {M}\limits _{\begin{array}{c} \approx \end{array}}}\nolimits ^\dag \mathop {M}\limits _{\begin{array}{c} \approx \end{array}})^* = \mathop {\mathop {M}\limits _{\begin{array}{c} \approx \end{array}}}\nolimits ^\dag \mathop {M}\limits _{\begin{array}{c} \approx \end{array}}. \end{aligned}$$

In particular, if $$\mathop {M}\limits _{\begin{array}{c} \approx \end{array}}$$ has a linearly independent columns, it holds that $$\mathop {\mathop {M}\limits _{\begin{array}{c} \approx \end{array}}}\nolimits ^\dag = (\mathop {\mathop {M}\limits _{\begin{array}{c} \approx \end{array}}}\nolimits ^\dag \mathop {M}\limits _{\begin{array}{c} \approx \end{array}})^{-1} \mathop {\mathop {M}\limits _{\begin{array}{c} \approx \end{array}}}\nolimits ^*$$.

#### Dynamic mode decomposition (DMD)

Given a data set in a form of a time series data as follows:

$$\begin{aligned} \mathop {T}\limits _{\begin{array}{c} \approx \end{array}} = \{ {\mathop {\mathop {u}\limits _{\sim }}\nolimits _0}, \mathop {\mathop {u}\limits _{\sim }}\nolimits _1, \ldots , \mathop {\mathop {u}\limits _{\sim }}\nolimits _{m-1}, \mathop {\mathop {u}\limits _{\sim }}\nolimits _m \} \in {\mathbb {C}}^{n \times (m+1)}, \end{aligned}$$

where $\mathop {\mathop {u}\limits _{\sim }}\nolimits _k$ stands for the $k$th snapshot of the data set for $k \ge 0$ with $m+1$ being the last entry of the data set, we let $$\mathop {X}\limits _{\begin{array}{c} \approx \end{array}}$$ and $$\mathop {Y}\limits _{\begin{array}{c} \approx \end{array}}$$ denote the followings:

$$\begin{aligned} \mathop {X}\limits _{\begin{array}{c} \approx \end{array}} = \{ \mathop {\mathop {u}\limits _{\sim }}\nolimits _0, \mathop {\mathop {u}\limits _{\sim }}\nolimits _1, \ldots , \mathop {\mathop {u}\limits _{\sim }}\nolimits _{m-1}\} \quad \text{ and } \quad \mathop {Y}\limits _{\begin{array}{c} \approx \end{array}} = \{ \mathop {\mathop {u}\limits _{\sim }}\nolimits _1, \mathop {\mathop {u}\limits _{\sim }}\nolimits _1, \ldots , \mathop {\mathop {u}\limits _{\sim }}\nolimits _{m}\}. \end{aligned}$$

We shall briefly review the general description of the dynamic mode decomposition (DMD) applied for $$\mathop {T}\limits _{\begin{array}{c} \approx \end{array}}$$. For clarity, we assume an ordered sequence of data separated by a constant sampling time $\Delta t$. The idea of DMD lies at the assumption that there exists a linear operator $$\mathop {A}\limits _{\begin{array}{c} \approx \end{array}}$$ that connects at least, approximately two data $\mathop {\mathop {u}\limits _{\sim }}\nolimits _k$ and its subsequent data $\mathop {\mathop {u}\limits _{\sim }}\nolimits _{k+1}$ for all $k \ge 0$, that is

1 $$\begin{aligned} \mathop {\mathop {u}\limits _{\sim }}\nolimits _{k+1} \approx \mathop {A}\limits _{\begin{array}{c} \approx \end{array}} \mathop {\mathop {u}\limits _{\sim }}\nolimits _k, \quad \forall k \ge 0 \quad \text{ equivalently } \quad \mathop {Y}\limits _{\begin{array}{c} \approx \end{array}} \approx \mathop {A}\limits _{\begin{array}{c} \approx \end{array}}\mathop {X}\limits _{\begin{array}{c} \approx \end{array}}. \end{aligned}$$

The ambiguity in the approximation $\approx$ will be clarified by defining $$\mathop {A}\limits _{\begin{array}{c} \approx \end{array}} = \mathop {Y}\limits _{\begin{array}{c} \approx \end{array}}\mathop {\mathop {X}\limits _{\begin{array}{c} \approx \end{array}}}\nolimits ^\dag $$ or as the solution to the following optimization problem:

2 $$\begin{aligned} \mathop {A}\limits _{\begin{array}{c} \approx \end{array}} = \mathop {\hbox {arg min}}\limits _{\mathop {C}\limits _{\begin{array}{c} \approx \end{array}}} \Vert \mathop {Y}\limits _{\begin{array}{c} \approx \end{array}} - \mathop {C}\limits _{\begin{array}{c} \approx \end{array}} \mathop {X}\limits _{\begin{array}{c} \approx \end{array}}\Vert _F, \end{aligned}$$

where $\Vert \cdot \Vert _F$ is the Frobenius norm. We note that the operator $$\mathop {A}\limits _{\begin{array}{c} \approx \end{array}}$$ is a type of dynamic operator that relates two consecutive data set. The goal of the dynamic mode decomposition is to extract the dynamic characteristic of $$\mathop {A}\limits _{\begin{array}{c} \approx \end{array}}$$, not directly to construct the mapping $$\mathop {A}\limits _{\begin{array}{c} \approx \end{array}}$$. More precisely, DMD obtains spectrums or spatial–temporal characteristics of the dynamical process described by $$\mathop {A}\limits _{\begin{array}{c} \approx \end{array}}$$. We note that the spectrums can be used to completely construct the action of the operator $$\mathop {A}\limits _{\begin{array}{c} \approx \end{array}}$$ if needs arise.

The essential algorithmic background lies in singular value decomposition of data, $$\mathop {X}\limits _{\begin{array}{c} \approx \end{array}}$$ and the relationship between eigen-pairs of $$\mathop {A}\limits _{\begin{array}{c} \approx \end{array}}$$ and its representation in principal component modes (see Lemma 1 and Lemma 2, in Supplementary note for Method). These are used to obtain the standard dynamic mode decomposition algorithm, as provided in Algorithm 1^51^.
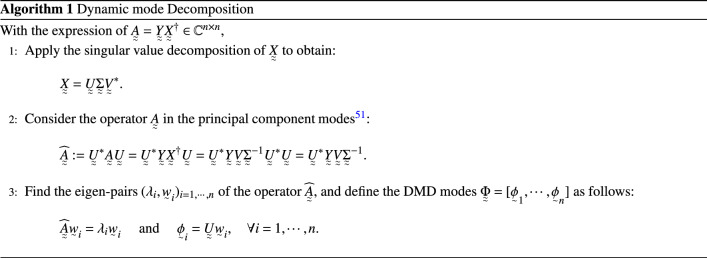
 Generally, the data analysis can be accomplished through the dynamic modes and eigenvalues, as given as $(\lambda _i, \mathop {\mathop {\phi }\limits _{\sim }}\nolimits _i, )_{i=1,\cdots ,n}$. We remark that $\{\mathop {\mathop {\phi }\limits _{\sim }}\nolimits _i\}_{i=1,\cdots ,n}$’s are called the DMD modes or mode vectors and they provide a rich set of information, especially spatial information about the data set^25^. For example, the modulus of the element of the mode vector provides measure of the spatial region’s participation for that mode. On the other hand, the eigenvalues $\{\lambda _i\}_{i=1,\cdots ,n}$ are relevant to the time evolution of the data sets and thus, they contain temporal information.

#### Linearity, consistency, and CwDMD

A loophole in DMD lies in that DMD spectrums are found for an approximate dynamic operator $$\mathop {A}\limits _{\begin{array}{c} \approx \end{array}}$$ for the data set $$\mathop {T}\limits _{\begin{array}{c} \approx \end{array}}$$. It is very much ambiguous and completely unknown theoretically how much the error observed in Eq. (1) results in misleading data interpretation from DMD spectrums. This has been elaborated in Fig. 8 for further clarity. The desired DMD is then not to start with constructing DMD-spectrums for $$\mathop {A}\limits _{\begin{array}{c} \approx \end{array}}$$ that satisfies (1), but, to build DMD spectrums based on $$\mathop {A}\limits _{\begin{array}{c} \approx \end{array}}$$ that satisfies the following relationship:

3 $$\begin{aligned} \mathop {\mathop {u}\limits _{\sim }}\nolimits _{k+1} = \mathop {A}\limits _{\begin{array}{c} \approx \end{array}} \mathop {\mathop {u}\limits _{\sim }}\nolimits _k, \quad \forall 0 \le k \le m, \quad \text{ equivalently } \quad \mathop {Y}\limits _{\begin{array}{c} \approx \end{array}} = \mathop {A}\limits _{\begin{array}{c} \approx \end{array}}\mathop {X}\limits _{\begin{array}{c} \approx \end{array}}. \end{aligned}$$

Thus, we investigate the condition for the existence of an operator $$\mathop {A}\limits _{\begin{array}{c} \approx \end{array}}$$ that satisfies the Eq. (3). This is in fact dependent on the data set $$\mathop {T}\limits _{\begin{array}{c} \approx \end{array}}$$. Namely, there must be a condition for $$\mathop {T}\limits _{\begin{array}{c} \approx \end{array}}$$, which leads to the existence of such an operator $$\mathop {A}\limits _{\begin{array}{c} \approx \end{array}}$$. Therefore, we introduce a notion of the linearity. Basically, we say that the data $$\mathop {T}\limits _{\begin{array}{c} \approx \end{array}}$$ is linear if and only if there exists an operator $$\mathop {A}\limits _{\begin{array}{c} \approx \end{array}} \in {\mathbb {C}}^{n\times n}$$ such that $$\mathop {Y}\limits _{\begin{array}{c} \approx \end{array}} = \mathop {A}\limits _{\begin{array}{c} \approx \end{array}} \mathop {X}\limits _{\begin{array}{c} \approx \end{array}}$$ (see the notion of linearity precisely defined for $$\mathop {T}\limits _{\begin{array}{c} \approx \end{array}}$$ in Definition 1 of Supplementary note). The compatibility condition is basically the condition for which the data $$\mathop {T}\limits _{\begin{array}{c} \approx \end{array}}$$ is linear. We remark that a relevant notion that states the Eq. (3) for a particular $$\mathop {A}\limits _{\begin{array}{c} \approx \end{array}}$$ of the form $$\mathop {A}\limits _{\begin{array}{c} \approx \end{array}} = \mathop {Y}\limits _{\begin{array}{c} \approx \end{array}}\mathop {\mathop {X}\limits _{\begin{array}{c} \approx \end{array}}}\nolimits ^\dag $$ for the data $$\mathop {T}\limits _{\begin{array}{c} \approx \end{array}}$$ has been provided by Tu et al. in^32^, i.e., a notion of linear consistency, stating that the null space of $$\mathop {X}\limits _{\begin{array}{c} \approx \end{array}}$$ is contained in that of $$\mathop {Y}\limits _{\begin{array}{c} \approx \end{array}}$$ $$({\mathcal {N}}(\mathop {X}\limits _{\begin{array}{c} \approx \end{array}}) \subset {\mathcal {N}}(\mathop {Y}\limits _{\begin{array}{c} \approx \end{array}}))$$ (see the notion of linear consistency defined for $$\mathop {T}\limits _{\begin{array}{c} \approx \end{array}}$$ in Definition 2 and also Theorem 1 of Supplementary note). We remark that the linearity is much more intuitive and general than the linear consistency. The notion of the linearity is a certain extension of the existence of line connecting two points in two dimensional Euclidean space consisting of one spatial dimension and one temporal dimension. On the other hand, we observe that these two concepts; linearity and linear consistency are in fact equivalent. Namely, the linear consistency of $$\mathop {T}\limits _{\begin{array}{c} \approx \end{array}}$$ holds if and only if the linearity of $$\mathop {T}\limits _{\begin{array}{c} \approx \end{array}}$$ holds (see Theorem 2 in Supplementary note for detailed proof). In another words, nonlinear data is inconsistent and inconsistent data is nonlinear. This equivalency is remarkable since these two concepts can be used to derive so-called the compatibility condition, which can be used to easily verify the linearity of $$\mathop {T}\limits _{\begin{array}{c} \approx \end{array}}$$. Note that the linear consistency condition provides an important algebraic condition for the data being linear. However, authors find it difficult to verify that condition in general.

The concept of compatibility is based on the observation that the data $$\mathop {T}\limits _{\begin{array}{c} \approx \end{array}}$$ being linear is relevant to the balance between spatial and temporal resolutions. As mentioned, for example, in one spatial dimension, only two points (two temporal data) can be connected in general by a line, unless data consisting of more than two points are collinear. Its extension for higher dimensional case can be understood as a simple inequality: $m \le n$. More precisely, the compatibility condition can be stated as follows:

##### **Definition**

(*Compatibility Condition*) Compatibility condition is the balance between to the balance between temporal and spatial resolutions, i.e., a data set $$\mathop {T}\limits _{\begin{array}{c} \approx \end{array}}$$ with the temporal resolution $m+1$ and spatial resolution *n* have the relationship that $m \le n$.

Note that for $m > n$, $$\mathop {T}\limits _{\begin{array}{c} \approx \end{array}}$$ will be in general inconsistent unless it is linear. The compatibility condition is stated to cover very general situations for which DMD can have a meaningful usage. We can show that under the compatibility condition, DMD will provide meaningful results with probability one. To be more precise, we note that the consistency can be easily understood in terms of the linear independency of the data $$\mathop {X}\limits _{\begin{array}{c} \approx \end{array}}$$, i.e., the linear independency of $$\mathop {X}\limits _{\begin{array}{c} \approx \end{array}}$$ implies the consistency of $$\mathop {T}\limits _{\begin{array}{c} \approx \end{array}}$$ and this can in particular, remove the trivial case that any column of $$\mathop {X}\limits _{\begin{array}{c} \approx \end{array}}$$ is the zero vector. Theoretically, it is established that if $$\mathop {T}\limits _{\begin{array}{c} \approx \end{array}}$$ satisfies the compatibility condition, then almost all $$\mathop {X}\limits _{\begin{array}{c} \approx \end{array}} \in {\mathbb {C}}^{n\times m}$$ with $m \le n$ will consist of columns which are linearly independent^52,53^. This means that $${\mathcal {N}}(\mathop {X}\limits _{\begin{array}{c} \approx \end{array}}) = \{\mathop {0}\limits _{\begin{array}{c} \approx \end{array}}\}$$. Therefore, the data set $$\mathop {T}\limits _{\begin{array}{c} \approx \end{array}}$$ is linear. The compatibility condition thus implies the consistency with probability one. Thus, the compatibility condition implies that the linearity of the data $$\mathop {T}\limits _{\begin{array}{c} \approx \end{array}}$$ is almost always guaranteed in case $m \le n$, which then leads to the meaningful DMD results.

In a very much rare case, when the consistency breaks under the compatibility condition, one can provide a small (arbitrarily small) perturbation to obtain $$\mathop {T_\varepsilon }\limits _{\begin{array}{c} \approx \end{array}} \in {\mathbb {C}}^{n\times (m+1)}$$, which is proven to result in a linear data^54^. Namely, for $m \le n$, let $$\mathop {X_\varepsilon }\limits _{\begin{array}{c} \approx \end{array}} \in {\mathbb {C}}^{n \times m}$$ consist of first *m* columns of $$\mathop {T_\varepsilon }\limits _{\begin{array}{c} \approx \end{array}}$$. Then we consider $$\widetilde{\mathop {X_\varepsilon }\limits _{\begin{array}{c} \approx \end{array}}} \in {\mathbb {C}}^{m\times m}$$ obtained from $$\mathop {X_\varepsilon }\limits _{\begin{array}{c} \approx \end{array}}$$ by chopping off all rows underneath $m$th row of $$\mathop {X_\varepsilon }\limits _{\begin{array}{c} \approx \end{array}}$$. This square matrix can be proven to be diagonalizable^52,54^, i.e., it consists of linear independent columns and thus the columns of $$\mathop {X_\varepsilon }\limits _{\begin{array}{c} \approx \end{array}}$$ is linearly independent. In view of the spatio-temporal analysis of the data, arbitrarily small perturbation will not change the result significantly. Furthermore, theoretically, such arbitrarily small perturbation will not affect the computation of the DMD-spectrums if they are in particular, Gaussian^55,56^. We remark that our data is generally very nice, i.e., whenever we choose $m \le n$, the data set $$\mathop {T}\limits _{\begin{array}{c} \approx \end{array}}$$ is always linear consistent and so, no perturbation was needed.

We are in a position to introduce our new algorithm, so-called a compatible window-wise dynamic mode decomposition (CwDMD). Our observation is that for $m > n$, $$\mathop {T}\limits _{\begin{array}{c} \approx \end{array}}$$ will be in general inconsistent unless it is linear. As such, the direct and reliable DMD analysis of large time series data is not feasible in general. The strategy is to choose an adequate set of representative subdomains called windows, each containing a moderate size of time-series data that satisfies the compatibility. The total size-times duration of all the windows serving a given system depends only on local situations that can arise in the full time series data. For example, Fig. 2, A shows a class of windows for the COVID-19 data in South Korea. Namely, given a data set $\{ \mathop {\mathop {u}\limits _{\sim }}\nolimits _0, \mathop {\mathop {u}\limits _{\sim }}\nolimits _1, \ldots , \mathop {\mathop {u}\limits _{\sim }}\nolimits _k, \ldots , \mathop {\mathop {u}\limits _{\sim }}\nolimits _m \}$, we consider the following windows that are consistent:

$$\begin{aligned} (\mathop {X_k}\limits _{\begin{array}{c} \approx \end{array}}, \mathop {Y_k}\limits _{\begin{array}{c} \approx \end{array}}), \text{ with } \mathop {X_k}\limits _{\begin{array}{c} \approx \end{array}} := \{ \mathop {u_{_{k_s}}}\limits _{\sim }, \ldots , \mathop {u_{_{k_e-1}}}\limits _{\sim } \} \quad \text{ and } \quad \mathop {Y_k}\limits _{\sim } := \{ \mathop {u_{_{k_s +1}}}\limits _{\sim }, \ldots , \mathop {u_{_{k_e}}}\limits _{\sim } \}. \end{aligned}$$

for which $$\mathop {X_k}\limits _{\begin{array}{c} \approx \end{array}}$$ and $$\mathop {Y_k}\limits _{\begin{array}{c} \approx \end{array}}$$ are consistent for $k = 0, 1, \ldots , \ell$. The compatible window-wise dynamic mode decomposition is to apply the dynamic mode decomposition locally for each compatible window $$(\mathop {X_k}\limits _{\begin{array}{c} \approx \end{array}}, \mathop {Y_k}\limits _{\begin{array}{c} \approx \end{array}})$$. Note that these windows can be constructed so that they may overlap or non-overlap depending on the situations. Therefore, choices of window can be made without too much restriction other than the condition of compatibility. This can be summarized as in the Algorithm 2.

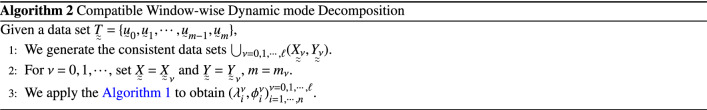



#### Data fitting, dimensional reduction, frequency and phase analysis

In this section, we discuss the data fitting using the DMD operator and choice of modes for the dimensional reduction and their uses for the phase analysis of each window. Throughout this section, we assume that $$\mathop {T}\limits _{\begin{array}{c} \approx \end{array}} \in {\mathbb {C}}^{n \times (m+1)}$$ is consistent and the DMD operator $$\mathop {A}\limits _{\begin{array}{c} \approx \end{array}}$$ is given in terms of eigen-pairs $(\lambda _i,\mathop {\mathop {\phi }\limits _{\sim }}\nolimits _i)_{i=1,\cdots ,n}$. We would also like to mention that the precise action of the operator $$\mathop {A}\limits _{\begin{array}{c} \approx \end{array}}$$ may not be found solely from these eigenspectrums. Namely, the data $$\mathop {X}\limits _{\begin{array}{c} \approx \end{array}}$$ has to be represented in terms of DMD modes, which requires to solve certain optimization problem. In a prior work, this has been accomplished by taking into account the whole data $$\mathop {X}\limits _{\begin{array}{c} \approx \end{array}}$$. We shall show that this can be done taking into account any single snapshot data in $$\mathop {X}\limits _{\begin{array}{c} \approx \end{array}}$$ under the consistency condition, thereby achieving a significant computational reduction. We begin our discussion with the fact that almost all complex matrices over complex fields are diagonalizable^52,54^. Namely, geometric and algebraic multiplicities of almost all complex matrices over complex fields are identical. This means that the DMD modes make a full set of eigenvectors for almost all data set satisfying the compatibility. Some list of a couple of equivalent conditions to the fact that algebraic and geometric multiplicities agree for a matrix $$\mathop {A}\limits _{\begin{array}{c} \approx \end{array}} \in {\mathbb {C}}^{n\times n}$$ can be found at^57^ and Theorem 3 in Supplementary note. Therefore, in general, we have that ${\mathbb {C}}^{n} = {\mathrm{span}} \{ \mathop {\mathop {\phi }\limits _{\sim }}\nolimits _i \}_{i=1,\cdots ,n}$. Having a full set of eigenvectors of $$\mathop {A}\limits _{\begin{array}{c} \approx \end{array}}$$, we can represent for example, the data $\mathop {\mathop {u}\limits _{\sim }}\nolimits _\eta$ of $$\mathop {T}\limits _{\begin{array}{c} \approx \end{array}}$$ with $0 \le \eta \le m+1$, as follows:

$$\begin{aligned} \mathop {\mathop {u}\limits _{\sim }}\nolimits _\eta = \sum _{i = 1}^n \alpha _i \mathop {\mathop {\phi }\limits _{\sim }}\nolimits _i \quad \text{ or } \quad \mathop {\alpha }\limits _{\sim } = \mathop {\mathop {\Phi }\limits _{\begin{array}{c} \approx \end{array}}}\nolimits ^{-1} \mathop {u_\eta }\limits _{\sim }, \end{aligned}$$

where $$\mathop {\Phi }\limits _{\begin{array}{c} \approx \end{array}} = [\mathop {\mathop {\phi }\limits _{\sim }}\nolimits _1 \, \ldots \, \mathop {\mathop {\phi }\limits _{\sim }}\nolimits _n]$$. With $\mathop {\alpha }\limits _{\sim }$ given above, we can obtain the action of the DMD operator $$\mathop {A}\limits _{\begin{array}{c} \approx \end{array}}$$ as follows: for $-\eta \le k \le -\eta + m + 1$,

4 $$\begin{aligned} \mathop {\mathop {u}\limits _{\sim }}\nolimits _k = \sum _{i=1}^n \alpha _i e^{k \, \mathfrak {R}{(\log (\lambda _i))}} e^{ \hat{i} k \mathfrak {I}{(\log (\lambda _i))}} \mathop {\mathop {\phi }\limits _{\sim }}\nolimits _i, \end{aligned}$$

where $\hat{i}$ is the pure imaginary number such that $\hat{i}^2 = -1$. We remark that it is standard to choose $\eta = 0$, which is also our choice. Oftentimes DMD is argued to be biased to the initial data^24^, our observation is that it is not really the case, for the consistent data. We recall that the framework of the optimized DMD^22^ is also designed to obtain the same $\mathop {\alpha }\limits _{\sim }$ for fitting, $$\mathop {X}\limits _{\begin{array}{c} \approx \end{array}}$$, by solving the following optimization problem:

$$\begin{aligned} \mathop {\alpha }\limits _{\sim } = \mathop {\hbox {arg min}}\limits _{\mathop {\mu }\limits _{\sim } = (\mu _i)_{i=1,\cdots ,n}} \left\| \mathop {X}\limits _{\begin{array}{c} \approx \end{array}} - \mathop {\Phi }\limits _{\begin{array}{c} \approx \end{array}} \mathop {D_{\mu }}\limits _{\begin{array}{c} \approx \end{array}} \mathop {V_{m-1}}\limits _{\begin{array}{c} \approx \end{array}} \right\| _{F}, \end{aligned}$$

where

$$\begin{aligned} \mathop {D_\mu }\limits _{\begin{array}{c} \approx \end{array}} = {\mathrm{diag}} (\mu ) \quad \text{ and } \quad \mathop {V_m}\limits _{\begin{array}{c} \approx \end{array}} = \left( \begin{array}{ccccc} 1 &{} \lambda _1 &{} \lambda _1^2 &{} \cdots &{} \lambda _1^m \\ 1 &{} \lambda _2 &{} \lambda _2^2 &{} \cdots &{} \lambda _2^m \\ \vdots &{} \vdots &{} \vdots &{} \ddots &{} \vdots \\ 1 &{} \lambda _n &{} \lambda _n^2 &{} \cdots &{} \lambda _n^m. \end{array} \right) \end{aligned}$$

It is clear that the consistency of data leads to a significant reduction of the computational effort.

We now can consider a discrete to continuous extension of the action of DMD operator. We remark that from the discrete represent of $\mathop {\mathop {u}\limits _{\sim }}\nolimits _k$ in (4), a continuous extension can be achieved as follows: for all $t \ge t_0 = 0$,

5 $$\begin{aligned} \mathop {u}\limits _{\sim }(t) := \sum _{i=1}^n \alpha _i (\lambda _i)^{t-t_0} \mathop {\mathop {\phi }\limits _{\sim }}\nolimits _i = e^{(t - t_0) \, \mathfrak {R}{(\log (\lambda _i))}} e^{ \hat{i} (t -t_0) \mathfrak {I}{(\log (\lambda _i))}} \mathop {\mathop {\phi }\limits _{\sim }}\nolimits _i. \end{aligned}$$

We now discuss the mode choice for the phase analysis, which will be used to obtain the dimensional reduction of the data. The most natural guide to choose the important DMD mode is to find the DMD mode which contributes most significantly to the data both temporally and spatially. This leads us to choose the index of DMD mode for which the following quantity, product of the temporal and spatial contribution in each window is maximized:

6 $$\begin{aligned} {\mathrm{arg}} \left\{ \max _k \{ |\lambda _k|^p \Vert \alpha _k \mathop {\mathop {\phi }\limits _{\sim }}\nolimits _k\Vert _F, 1 \le k \le n. \} \right\} , \end{aligned}$$

where *p* is the temporal resolutions for the window. We call the quantity $|\lambda _k|^p \Vert \alpha _k \mathop {\mathop {\phi }\limits _{\sim }}\nolimits _k\Vert _F$ the power of the $k$th DMD mode and observe that in general one or two dominant powers exist. These are then chosen to form a dimensionally reduced data. For example, $\mathop {\mathop {\phi }\limits _{\sim }}\nolimits _k$ is the DMD mode whose power is the largest. Then it is used to form a dimensionally reduced data: for all $t \ge t_0 = 0$,

7 $$\begin{aligned} \mathop {{\widetilde{u}}}\limits _{\sim }(t) = \alpha _k (\lambda _k)^{t-t_0} \mathop {\mathop {\phi }\limits _{\sim }}\nolimits _k = e^{(t - t_0) \, \mathfrak {R}{(\log (\lambda _k))}} e^{ \hat{i} (t -t_0) \mathfrak {I}{(\log (\lambda _k))}} \mathop {\mathop {\phi }\limits _{\sim }}\nolimits _k, \end{aligned}$$

which is used for the data interpretation such as phases and magnitudes. In literature, DMD modes are chosen based on their norms or weighted norm by the corresponding DMD eigenvalues^32^. For example, the use of weighted norm by DMD eigenvalues, can be interpreted as to penalize spurious modes with large norms but quickly decaying contributions to the dynamics^29^. In our choice, we incorporate $\mathop {\alpha }\limits _{\sim }$, the coordinate of data in the frame of DMD modes as a special scale for DMD modes. These measurements are meaningful especially for highly nonlinear data, since coordinates given in terms of DMD modes can much affect the dynamics of data. We remark that the frequency of the solution for the mode *k*, can be defined through $\mathfrak {I}{(\log (\lambda _k))}/2\pi$ and thus the period is given by the reciprocal of the frequency. The identified DMD mode can be categorized as periodic, growing or decaying modes depending on the magnitude of $\lambda _k$. Namely, for eigenvalues on (or close), outside or inside the unit circle, the corresponding modes are considered as oscillatory, growing, and decaying modes, respectively. In the present work, we give a tolerance $\epsilon = 5.E{-}2$ and denote $N_o = \{ i : ||\lambda _i| - 1| \le \epsilon \}$, $N_g = \{ i : |\lambda _i| > 1 + \epsilon \}$, $N_d = \{ i : |\lambda _i| < 1 - \epsilon \}$ by the set of oscillatory modes, the set of growing modes, and the set of decaying modes, respectively. We first select the DMD modes of large powers, and then measure the magnitude of its eigenvalues and determine whether they are oscillatory, growing or decaying mode.

## Supplementary Information

Supplementary Information.

## Data Availability

The map of South Korea was obtained in the form of a shapefile from the website of the Statistical Geographic Information Service (panel A of Fig. 2)^58^. Population, area, and density by the 17 regions as of April 9, 2021, were obtained from the website of e-indicatior in South Korea (panel B of Fig. 2)^59^. The daily incidence of COVID-19 by regions from January 20, 2020, to May 10, 2021, was obtained on the website of Seoul National University Asia Regional Information Center (SNUARIC) (panel B of Fig. 2)^60^.
