# Supplementary material for: Discovering spatiotemporal patterns of COVID-19 pandemic in South Korea
Source: Sci Rep. 2021 Dec 28;11:24470. doi: 10.1038/s41598-021-03487-2 (PMC8714822; doi:10.1038/s41598-021-03487-2)
Supplement: Supplementary file 1 — Supplementary Information. [file 41598_2021_3487_MOESM1_ESM.pdf]

Supplementary Information: Discovering spatiotemporal patterns of COVID-19 pandemic in South Korea

Sungchan Kim[1], **Minseok Kim**[1], **Sunmi Lee**[1,*], **and Young Ju Lee**[2,*]

[1]Department of Applied Mathematics, Kyung Hee University, Republic of Korea
[2]Department of Mathematics, Texas State University, TX, USA
[*]sunmilee@gkhu.ac.kr (S. Lee), yjlee@txstate.edu (YJ Lee)

Supplementary note for Method

This section is to provide formal Lemmata and Theorems used in the section for Method in the main text.

Lemma 1 (Singular Value Decomposition)**.** *Let $\underset{\approx}{M} \in \mathbb{C}^{n\times m}$. Then, there exists a factorization given as follows:*

$$\underset{\approx}{M} = \underset{\approx}{U}\underset{\approx}{\Sigma}\underset{\approx}{V}^*, \tag{1}$$

where $\underset{\approx}{U} \in \mathbb{C}^{n\times n}$, $\underset{\approx}{\Sigma} \in \mathbb{C}^{n\times m}$ and $\underset{\approx}{V} \in \mathbb{C}^{m\times m}$ with

$$\underset{\approx}{U}^*\underset{\approx}{U} = \underset{\approx}{\delta} \in \mathbb{C}^{n\times n} \quad \text{and} \quad \underset{\approx}{V}^*\underset{\approx}{V} = \underset{\approx}{\delta} \in \mathbb{C}^{m\times m}.$$

Lemma 2. *Let $\underset{\approx}{A} \in \mathbb{C}^{n\times n}$ and $\widehat{\underset{\approx}{A}} \in \mathbb{C}^{n\times n}$ be related through the following equation:*

$$\widehat{\underset{\approx}{A}} = \underset{\approx}{U}^*\underset{\approx}{A}\underset{\approx}{U},$$

where $\underset{\approx}{U} \in \mathbb{C}^{n\times n}$ be any unitary matrix. We assume that $(\lambda_i, \underset{\sim}{w}_i)_{i=1,\cdots,n}$ are eigen-pairs of $\widehat{\underset{\approx}{A}}$. Then $(\lambda_i, \underset{\sim}{\phi}_i)_{i=1,\cdots,n}$ makes eigen-pairs for $\underset{\approx}{A}$, where $\underset{\sim}{\phi}_i = \underset{\approx}{U}\underset{\sim}{w}_i$ for all i.

Proof. Let $(\lambda_i, \underset{\sim}{w}_i)_i$ be eigenpairs for $\widehat{\underset{\approx}{A}}$. Then from the relation that

$$\widehat{\underset{\approx}{A}} = \underset{\approx}{U}^*\underset{\approx}{A}\underset{\approx}{U},$$

we observe that for all i,

$$\lambda_i \underset{\sim}{w}_i = \widehat{\underset{\approx}{A}}\underset{\sim}{w}_i = \underset{\approx}{U}^*\underset{\approx}{A}\underset{\approx}{U}\underset{\sim}{w}_i.$$

This gives

$$\lambda_i \underset{\approx}{U}\underset{\sim}{w}_i = \underset{\approx}{A}\underset{\approx}{U}\underset{\sim}{w}_i,$$

which completes the proof. □

Definition 1 (Linear data)**.** *The data set $\underset{\approx}{T} = \{\underset{\sim}{u}_0, \cdots, \underset{\sim}{u}_m\} \in \mathbb{C}^{n\times(m+1)}$ is called linear if there exists a matrix $\underset{\approx}{A} \in \mathbb{C}^{n\times n}$ such that $\underset{\approx}{Y} = \underset{\approx}{A}\underset{\approx}{X}$, where $\underset{\approx}{X} = \{\underset{\sim}{u}_0, \cdots, \underset{\sim}{u}_{m-1}\}$ and $\underset{\approx}{Y} = \{\underset{\sim}{u}_1, \cdots, \underset{\sim}{u}_m\}$.*

Definition 2 (Linear consistency)**.** *The data set $\underset{\approx}{T} = \{\underset{\sim}{u}_0, \cdots, \underset{\sim}{u}_m\} \in \mathbb{C}^{n\times(m+1)}$ is called linear consistent if its subset data $\underset{\approx}{X} = \{\underset{\sim}{u}_0, \cdots, \underset{\sim}{u}_{m-1}\}$ and $\underset{\approx}{Y} = \{\underset{\sim}{u}_1, \cdots, \underset{\sim}{u}_m\}$, both in $\mathbb{C}^{n\times m}$ satisfy the relation that $\mathcal{N}(\underset{\approx}{X}) \subset \mathcal{N}(\underset{\approx}{Y})$.*

Theorem 1. *Let $\underset{\approx}{A} = \underset{\approx}{Y}\underset{\approx}{X}^\dagger \in \mathbb{C}^{n\times n}$. Then $\underset{\approx}{Y} = \underset{\approx}{A}\underset{\approx}{X}$ if and only if $\underset{\approx}{X}$ and $\underset{\approx}{Y}$ are linearly consistent.*

Proof. Assume that $\underset{\approx}{X}$ and $\underset{\approx}{Y}$ are not linearly consistent. There exists $\underset{\sim}{c} \in \mathcal{N}(\underset{\approx}{X})$ such that $\underset{\approx}{Y}\underset{\sim}{c} \neq \underset{\sim}{0}$. Then, it holds that $\underset{\approx}{A}\underset{\approx}{X} \neq \underset{\approx}{Y}$ for any $\underset{\approx}{A}$. Conversely, we assume that $\underset{\approx}{X}$ and $\underset{\approx}{Y}$ are linearly consistent. We then observe that

$$\underset{\approx}{Y} - \underset{\approx}{A}\underset{\approx}{X} = \underset{\approx}{Y} - \underset{\approx}{Y}\underset{\approx}{X}^\dagger\underset{\approx}{X} = \underset{\approx}{Y}(\underset{\approx}{\delta} - \underset{\approx}{X}^\dagger\underset{\approx}{X}).$$

On the other hand, $\underset{\approx}{\delta} - \underset{\approx}{X}^\dagger\underset{\approx}{X}$ is a orthogonal projection onto $\mathcal{N}(\underset{\approx}{X})$. Thus we arrive at $\underset{\approx}{Y} - \underset{\approx}{A}\underset{\approx}{X} = \underset{\approx}{0}$. This completes the proof. □

Theorem 2. *Let $\underset{\approx}{T} \in \mathbb{C}^{n\times(m+1)}$ be a given data set. It is linear consistent if and only if it is linear.*

Proof. Assume that two matrices $\underset{\approx}{X}$ and $\underset{\approx}{Y}$ are linear, i.e., there exists $\underset{\approx}{A} \in \mathbb{C}^{n\times n}$ such that $\underset{\approx}{Y} = \underset{\approx}{A}\underset{\approx}{X}$. It is clearly then that $\mathcal{N}(\underset{\approx}{X}) \subset \mathcal{N}(\underset{\approx}{Y})$. Therefore, they are linearly consistent. On the other hand, if $\underset{\approx}{T}$ is linearly consistent, then it is linear from Theorem 1. This completes the proof. □

Theorem 3. *Let $\underset{\approx}{A} \in \mathbb{C}^{n\times n}$ and λ be an eigenvalue of $\underset{\approx}{A}$. Then the following statements are equivalent:*

(1) $\mathbb{C}^n = \mathcal{N}(\underset{\approx}{A} - \lambda\underset{\approx}{\delta}) \oplus \mathcal{R}(\underset{\approx}{A} - \lambda\underset{\approx}{\delta})$.

(2) $\mathcal{N}(\underset{\approx}{A} - \lambda\underset{\approx}{\delta}) \cap \mathcal{R}(\underset{\approx}{A} - \lambda\underset{\approx}{\delta}) = \{\underset{\approx}{0}\}$.

(3) The geometric multiplicity and the algebraic multiplicity of λ are equal.

Supplementary figures

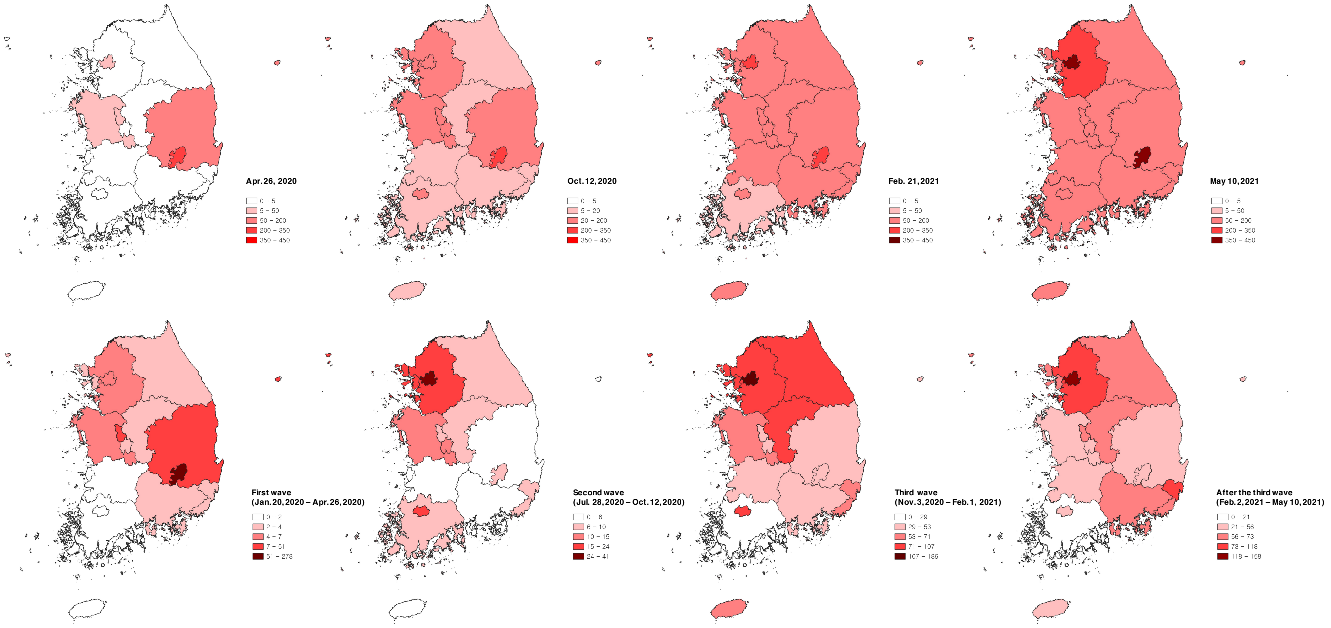

Figure S1. Spatial distributions of 17 regions are shown in each period. The top panels show the cumulative number of COVID-19 cases per 100,000 on the last day of each period while the bottom panels show the cumulative number of COVID-19 cases per 100,000 during each period.

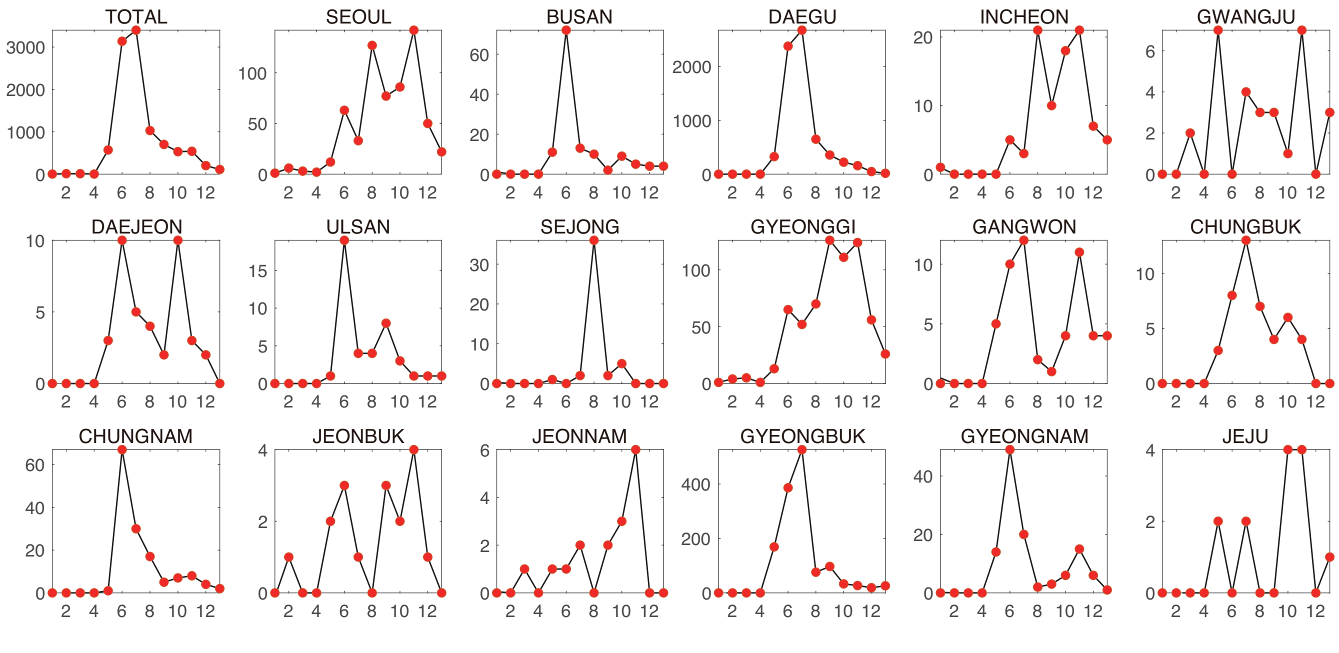

Figure S2. The fitted curves to reported data in the first wave. In each panel, red dots show the weekly cases data and bold line indicates the fitted curve.

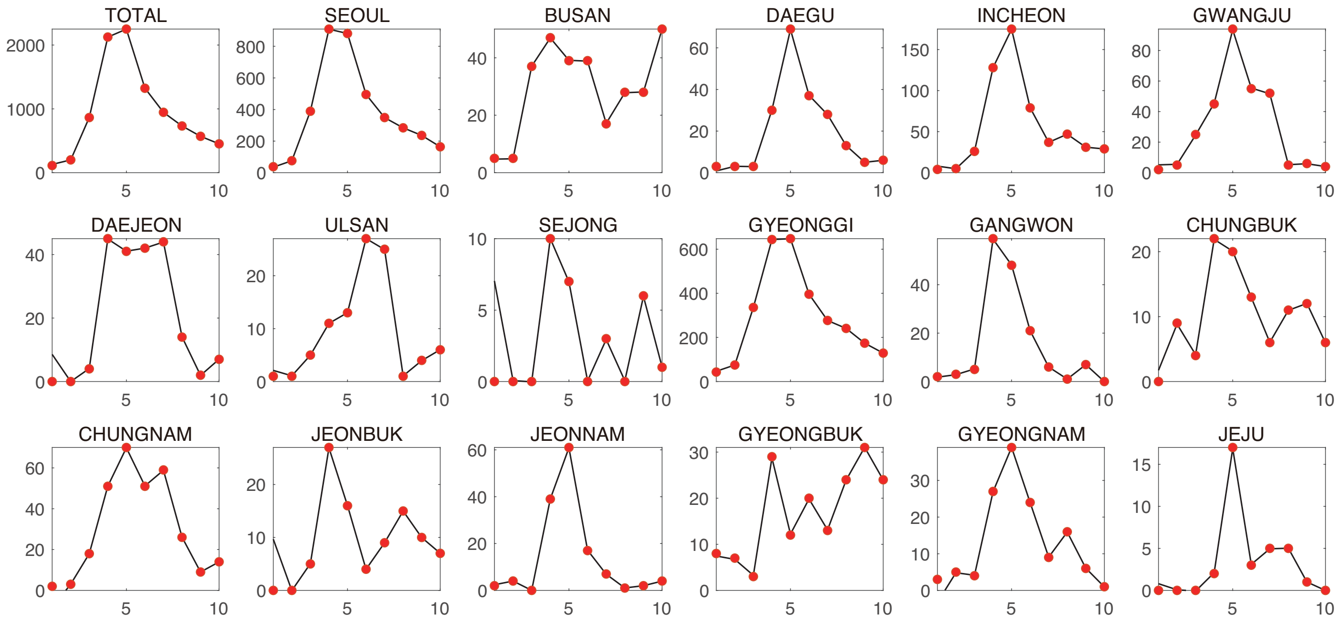

Figure S3. The fitted curves to reported data in the second wave. In each panel, red dots show the weekly cases data and bold line indicates the fitted curve.

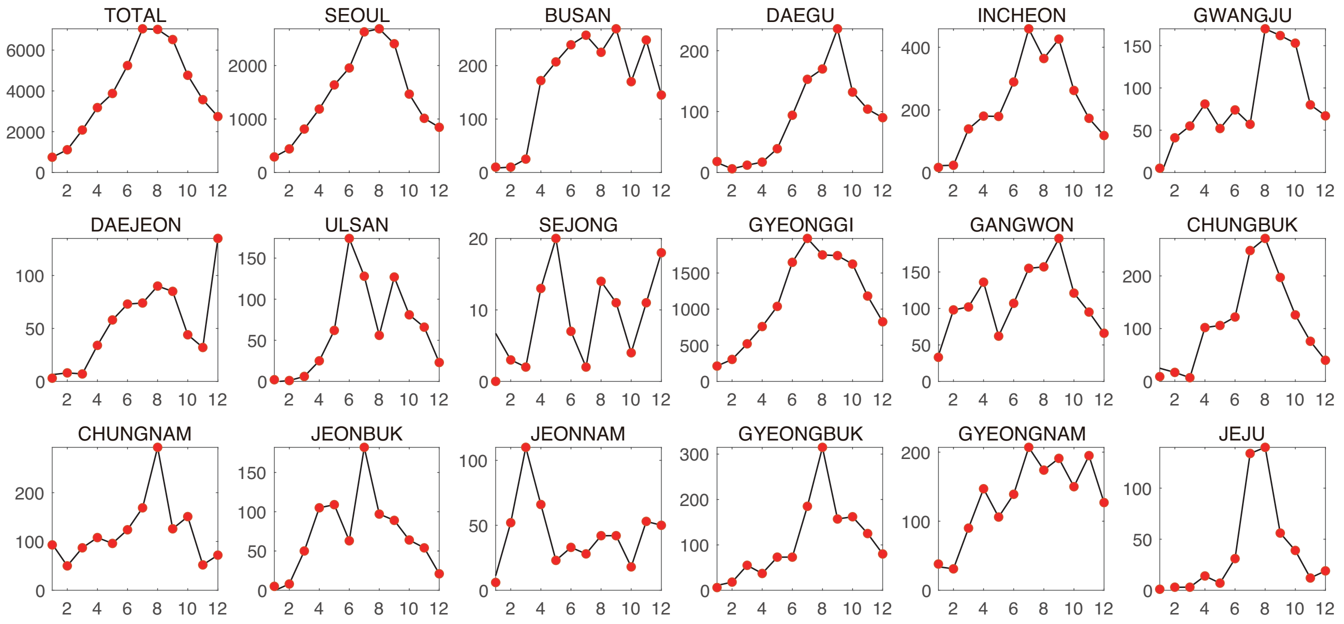

Figure S4. The fitted curves to reported data in the third wave. In each panel, red dots show the weekly cases data and bold line indicates the fitted curve.

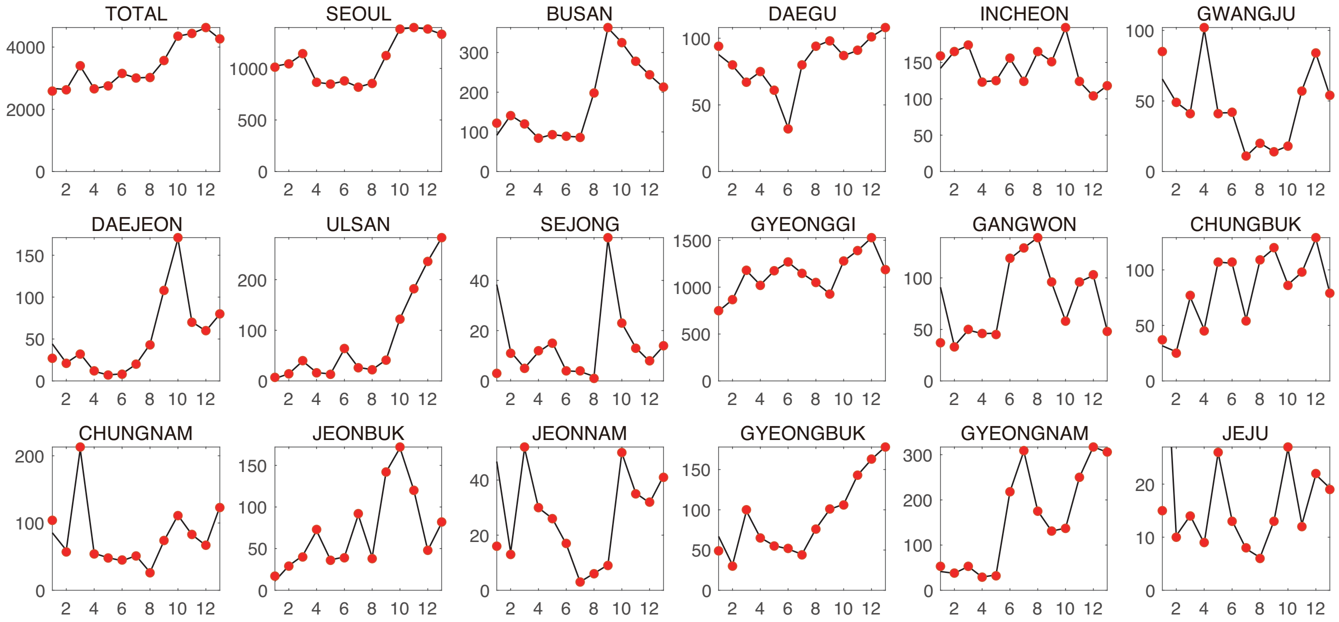

Figure S5. The fitted curves to reported data in the period after the third wave. In each panel, red dots show the weekly cases data and bold line indicates the fitted curve.
